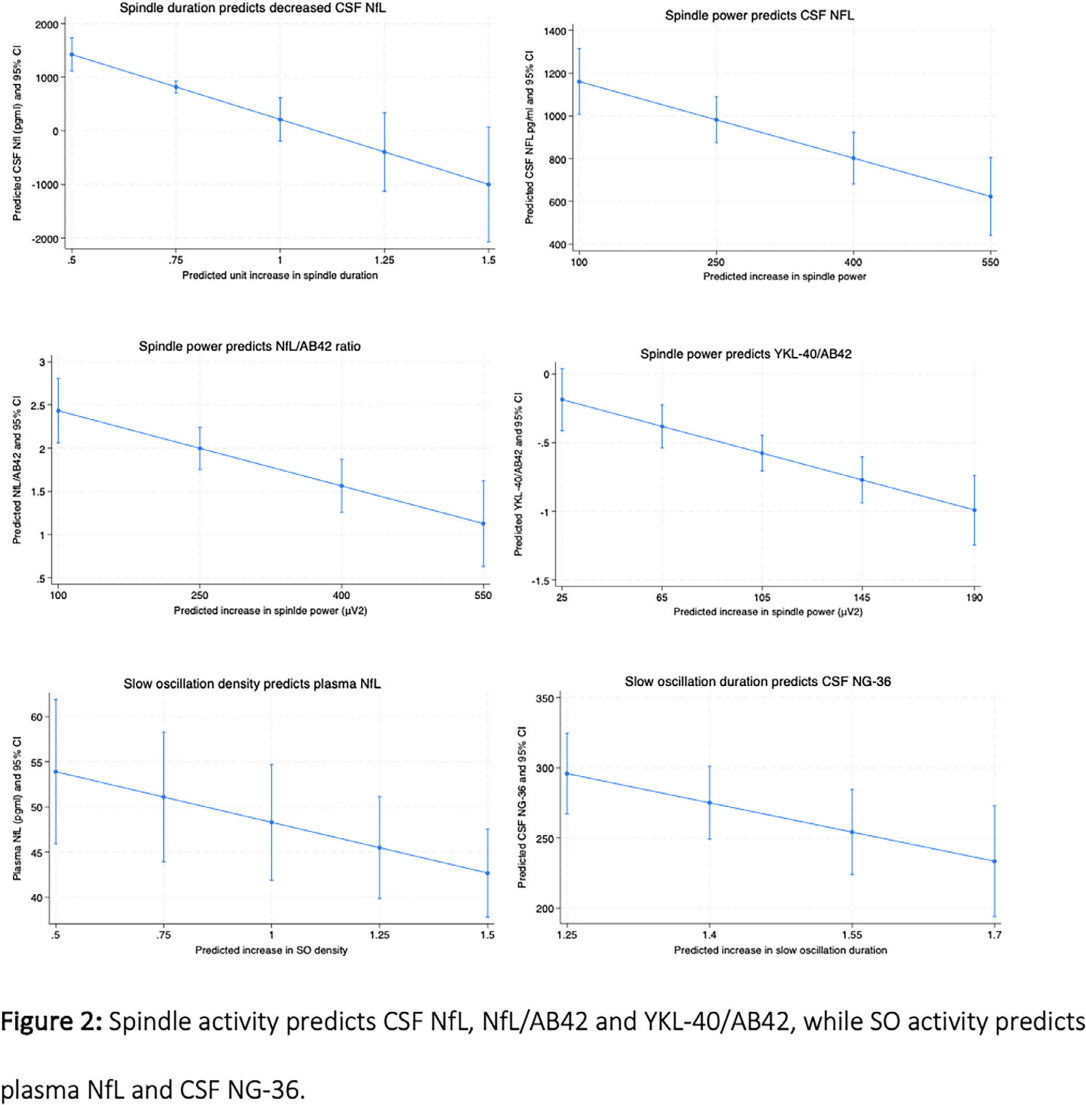# Sleep spindles and slow oscillations predict neurofilament‐light, neurogranin 36, Chitinase‐3‐like protein‐1 and cognition in mild to moderate Alzheimer's Disease

**DOI:** 10.1002/alz70856_107617

**Published:** 2026-01-09

**Authors:** Arsenio Paez, Shahla Bakian Dogaheh, Sam O Gillman, Anna Carnes, Farida Dakterzada, Ferran Barbe, Gerard Piñol‐Ripoll, Thien Thanh Dang‐Vu

**Affiliations:** ^1^ University of Oxford, Oxford, Oxfordshire, United Kingdom; ^2^ Centre de Recherche de l’Institut Universitaire de Gériatrie de Montréal, Montreal, QC, Canada; ^3^ Concordia University, Montreal, QC, Canada; ^4^ Hospital Universitari Santa Maria de Lleida, IRBLleida, Lleida, Spain; ^5^ Hospital Universitari Santa Maria LLeida, LLeida, Spain; ^6^ Institut de Recerca Biomédica de Lleida (IRBLLeida), Lleida, Spain; ^7^ Cognitive Disorder Unit, Hospital Universitari Santa Maria, Lleida, Spain; ^8^ Centre de recherche de l'Institut universitaire de gériatrie de Montréal, Montreal, QC, Canada

## Abstract

**Background:**

Sleep is essential for brain‐health, including clearance of β‐amyloid (Aβ), tau, and otherpromising diagnostic markers of neurodegeneration and progression in Alzheimer's Disease (AD): cerebrospinal fluid neurofilament‐light chain (NfL), neurogranin‐36 (NG‐36), and Chitinase‐3‐like protein‐1 (YKL‐40). However, it remains unclear which sleep characteristics predict these biomarkers or whether the biomarkers predict cognitive or neuropsychiatric decline after AD onset.

**Methods:**

Using data from a prospective cohort study of mild‐to‐moderate AD (*n* = 60, 30‐female, mean age 74.7), we analysed non‐rapid eye‐movement sleep spindles and slow oscillations (SO) at baseline and their associations with baseline NfL, YKl‐40, NG‐36, NfL/Aβ42, YKl‐40/Aβ42, and whether these biomarkers predict cognition and mental health from baseline to three‐years follow‐up.

Participants underwent baseline polysomnography (PSG) and cerebrospinal fluid draws for amyloid and tau, and neuropsychological assessment at baseline, 12, 24 and 36 months with the Mini‐Mental Status Examination (MMSE), and the Alzheimer's Disease AssessmentScale‐Cognitive Subscale (ADAS‐Cog) and Neuropsychiatric Inventory (NPI) at baseline and 12 months.

Spindle and SO detection were performed using in‐house, open‐source software packages developed at Concordia University. Associations between SO and spindle characteristics (duration, density, power, amplitude), biomarkers, and cognition from baseline to 36 months were investigated with false discovery rate‐adjusted robust regression controlling for age, sex, apnea‐hypopnea index.

**Results:**

We found previously unreported associations between spindle and SO characteristics, NfL, YKl‐40, NG‐36, NfL/Aβ42 (β=‐.0029, *p* = 0.001), YKl‐40/Aβ42 (β=0.0004, *p* = 0.003) and cognition in persons with AD. These biomarkers predicted worse cognitive performance (higher ADAS‐cog [β=2.28, *p* = 0.004], lower MMSE scores [β= ‐2.42, *p* = 0.01]) from baseline to 36‐months, and a significant increase in neuropsychiatric symptom severity (NPI β=16.93 *p* <0.001). NfL/Aβ42 mediated the effects of spindle activity on cognitive performance on the ADAS‐cog (*p* =  0.041) and MMSE (*p* = 0.0019). Biomarkers also moderated the relationships between spindle and SO activity on cognition, and spindles and SO moderated the relationships between these biomarkers and cognition.

**Conclusions:**

Our novel findings demonstrate that spindle and SO activity are associated with NfL, YKl‐40, and NG‐36, and cognitive decline, constituting predictive, non‐invasive biomarkers of neurodegeneration, cognition, and mental health in AD. They may thus provide novel treatment targets for delaying AD progression.